# Emerging technologies for salivaomics in cancer detection

**DOI:** 10.1111/jcmm.13007

**Published:** 2016-11-13

**Authors:** Karolina Elżbieta Kaczor‐Urbanowicz, Carmen Martín Carreras‐Presas, Tadeusz Kaczor, Michael Tu, Fang Wei, Franklin Garcia‐Godoy, David T. W. Wong

**Affiliations:** ^1^ Center for Oral/Head & Neck Oncology ResearchSchool of DentistryUniversity of California at Los AngelesLos AngelesCAUSA; ^2^ Faculty of Biomedical SciencesAdult's Dentistry DepartmentUniversidad Europea de MadridMadridSpain; ^3^ Faculty of Mechanical EngineeringDepartment of PhysicsKazimierz Pulaski University of Technology and Humanities in RadomRadomPoland; ^4^ Bioscience Research CenterCollege of DentistryUniversity of Tennessee Health Science CenterMemphisTNUSA

**Keywords:** salivary diagnostics, cancer, RNA‐Sequencing, point‐of‐care, liquid biopsy

## Abstract

Salivary diagnostics has great potential to be used in the early detection and prevention of many cancerous diseases. If implemented with rigour and efficiency, it can result in improving patient survival times and achieving earlier diagnosis of disease. Recently, extraordinary efforts have been taken to develop non‐invasive technologies that can be applied without complicated and expensive procedures. Saliva is a biofluid that has demonstrated excellent properties and can be used as a diagnostic fluid, since many of the biomarkers suggested for cancers can also be found in whole saliva, apart from blood or other body fluids. The currently accepted gold standard methods for biomarker development include chromatography, mass spectometry, gel electrophoresis, microarrays and polymerase chain reaction‐based quantification. However, salivary diagnostics is a flourishing field with the rapid development of novel technologies associated with point‐of‐care diagnostics, RNA sequencing, electrochemical detection and liquid biopsy. Those technologies will help introduce population‐based screening programs, thus enabling early detection, prognosis assessment and disease monitoring. The purpose of this review is to give a comprehensive update on the emerging diagnostic technologies and tools for the early detection of cancerous diseases based on saliva.

**Table:** 

• Introduction
• Emerging technologies for salivary diagnostics of cancer
– Point‐of‐care diagnostics
– RNA Sequencing
– Liquid biopsy
– Electromagnetic field‐based methods
– Electric field induced release and measurement method
• Conclusions

## Introduction

Saliva is a complex fluid that is composed of water, cells, debris, organic and inorganic molecules that may reflect the physiological state of an individual condition, since many of the componenets of the saliva also play an important role in processes taking part in distal portions of the body 1. Currently, approximately 40% of markers suggested for diseases such as cancer, cardiovascular disease and stroke can also be found in whole saliva 2. A biomarker can be defined as a measurable and quantifiable biological parameter that can serve either as an indicator for health, disease status, environmental exposure or pharmacological responses to a therapeutic intervention 3. Prognostic biomarkers are used as indicators of a benign or a malignant condition, whereas diagnostic biomarkers show the development of a cancer 4.

Siegel *et al*. reported that 1.7 million Americans are diagnosed with cancer every year and over 500,000 individuals do not survive the disease 5. Hence, a lot of efforts have been done to advance the field of salivary diagnostic technology, which is likely to revolutionize the way cancerous diseases will be diagnosed in the future 6.

To successfully translate research on salivary biomarkers to the chairside, biomarker studies should follow the principles laid out in the prospective‐specimen‐collection, retrospective‐blinded evaluation (PRoBE design). In this approach, samples are first collected prospectively from a cohort of target population prior to diagnosis. After examination of the patient, individuals with known diagnosis and control subjects will be selected randomly from the cohort and their specimens will be tested in a blinded study design 7. If applied with rigour and appropriate sampling of patient population, definitive validation of identified biomarkers can result in the Food and Drug Administration regulatory approval.

Despite the acceptance of salivary diagnostics for the detection of cancerous diseases, the absence of a mechanistic rationale in regards to the transmission of biomarkers between the distal tumour and the oral cavity poses a risk that has the potential of undermining saliva's value for the detection of tumour diseases. However, recent studies focused on exosome secretion and biogenesis have attempted to unravel this phenomenon 8, 9, 10.

## Emerging technologies for salivary diagnostics of cancer

### Point‐of‐care diagnostics

Point‐of‐care (POC) technologies are newly emerging methods, that when used in conjunction with biomarker identification, have the potential to be used in screening and non‐invasive diagnostics in a rapid and convenient fashion 11. Current diagnostic methods used for the detection of malignant cancers have also significant limitations such as low sensitivity and low specificity. They are time‐consuming, invasive, cost‐prohibitive, and complex to perform 12, 13. In addition, the long assay time may cause degradation of many important constituents in the patient samples before quantification can be made 14, 15. Therefore, before entering the clinical settings, these POC tests should be appropriately prepared *i.e*. to prove their validity, reliability, reproducibility and robustness 16.

There are several newly emerging technologies that integrate salivary diagnostics with microfluidics or micro/nanoelectromechanical systems (MEMS/NEMS). Microfluidics consist of manipulation of liquids at the microscale to miniaturize and automate many techniques that may normally require trained personnel with traditional laboratory equipment. MEMS/NEMS devices are composed of mechanical elements, sensors, actuators and electronics on a common silicon substrate developer through microfabrication technology. Those technologies enable users to measure proteins, DNA, transcripts (mRNA), electrolytes and small molecules in saliva 17, 18. Currently developed MEMS/NEMS platforms use a variety of techniques to perform detection, including electrochemical sensing 19, on‐chip reverse transcription polymerase chain reaction (RT‐PCR) 20, microsphere‐based optical fibre array 21, high‐throughput DNA microarray, surface plasmon resonance optical system 22 or microchip electrophoretic immunoassay 23.

Mishra *et al*. divided currently used technologies and devices according to the type of biomarker and cancer, as follows 13. The biomarker types are:

- Transcriptomic biomarkers: breast cancer (nanographene oxide‐polyethylene glycol methyl ether methacrylate with DNase 1 to detect microRNA‐10b and microRNA‐10a) 24, oral cancer (electrical controlled magnetic EC Sensor to detect microRNA‐200a) 25, prostate cancer [nano‐graphene oxide (nGO)/FAM‐anti‐miR‐21 to detect microRNA‐21 and nGO/Cy5‐anti‐miR‐141 to detect microRNA‐141] 26;
- Genomic biomarkers: oral cancer [electrochemical sensor using endonuclease target recycling amplification to detect oral cancer overexpressed 1 (ORAOV1) 27 or electric field‐induced release and measurement method for detection of epidermal growth factor receptor (EGFR) 28];
- Metabolomic biomarkers: oral cancer (wireless mouthguard ezymatic biosensor to detect uric acid 29 or lactic acid 30), gastric cancer (microfluidic optoelectronic 31 or graphene based‐antimicrobial peptides with passive detection of Helicobacter pylori 32);
- Proteomic biomarkers: liver cancer (surface enhanced Raman spectroscopy using optical nanoanntenas functionalized with aptamers for detection of MnSOD) 33, breast cancer [surface plasma resonance biosensor based on Au/ZnO thin films for carcinoma antigen 15‐3 (CA15‐3)] 34;
- Multiplex: silicon nanowire field effect transistor for IL‐8 and TNF‐α 35.

The newly developed POC tests for ‘lab‐on‐a‐chip’ allows detection of multiple biomarkers, thus facilitating the diagnosis of many human diseases at the same time 36.

In 2003, the University of California at Los Angeles (UCLA) Collaborative Oral Fluid Diagnostic Research Center was established with the major aim of developing the platform for using nanotechnology and microtechnology for detection of salivary proteins and genomic biomarkers. An integrated POC electrochemical multiplexing saliva‐based platform for oral cancer detection emerged 17, 37. This platform can detect both salivary proteins and nucleic acids as well as measure up to eight different biomarkers in a single test in less than 15 min. under ambient conditions. The salivary test in an Indian cohort of oral cancer saliva samples achieved 90% sensitivity and 90% specificity for both interleukin 8 (IL‐8) and IL‐8 protein messenger RNA (mRNA) 38. This method can potentially be used for screening and assessment of the risk for oral cancer, as well as identification of patients that may need a biopsy 37. Another saliva‐based molecular test, OraRisk^®^ human papilloma virus (HPV) with Reflex (Quest Diagnostics, Los Angeles, CA, USA) can determine the presence of HPV types associated with a high risk of developing oral cancer.

Emerging novel POC techniques used specifically for oncogenic mutation detection in clinical practice include: gold nanoparticle‐based mutation capture and naked‐eye visualization for the detection of single nucleotide polymorphism mutation 39, 40 or a combination of magnetic and gold nanoparticle methods to identify KRAS gene mutations 41. Another method – microfluidic platforms combine genetic analysis with microfluidic systems 40, 42, for example, an integrated microfluidic system for JAK2‐V617F mutation detection, present in various hematological malignancies 43.

Along with these recent scientific advancements, there is an emerging need to move salivary diagnostics out of the research lab into clinical practice. Point‐of‐care technologies can provide non‐invasive, rapid, easy and accurate measurements directly from saliva for monitoring different medical conditions including cancers 37.

### RNA sequencing

RNA‐Sequencing (RNA‐Seq) is a newly emerging high‐throughput method for performing transcriptome profiling by means of deep‐sequencing technologies. The transcriptome is composed of transcripts in a cell, and their quantity. It plays a crucial role for unravelling functional elements of the genome, the molecular components of cells, and also the mechanisms of normal development, physiology and pathology. While performing RNA Sequencing, RNA is converted to a library of cDNA fragments. Each molecule is sequenced, with or without amplification, resulting in the large number of reads, that are subsequently aligned either to a reference genome or to a transcriptome 44. RNA‐Seq has many advantages over the currently used DNA microarrays, *i.e*.: ability to detect transcripts and their isoforms, low background signal, increased dynamic range of expression, measurement of focal changes, splice variants, chimeric gene fusions and applicabilty to each species, *etc*. 44, 45.

Although RNA‐Seq of saliva is challenging, because of factors such as the difficulty of performing RNA isolation, stabilitization, RNA library construction, *etc*. 46, 47, 48, 49, the recent advancements in the field have resulted in the identification of various types of extracellular RNAs (exRNAs) such as: mRNAs and non‐coding RNAs (ncRNAs) including microRNAs (miRNAs), piwi‐interacting RNAs (piRNAs) and circular RNAs (circRNAs). Specifically, those ncRNAs are emerging regulators of oncogenesis and tumour progression. Because of their small size, they are more stable and less prone to degradation by ribonucleases (RNases) compared to mRNAs 15.

Currently, RNA Sequencing has a wide range of applications in cancer diagnostics. Our group at UCLA is in the process of developing salivary biomarkers for early detection of gastric cancer 50. This study involves comprehensive RNA‐Sequencing performed on 100 randomly selected gastric cancer saliva samples and 100 randomly selected non‐gastric cancer matched control subjects. Bioinformatic analysis of RNA‐Seq data has revealed various types of exRNAs in cell‐free saliva, including 127–418 miRNAs, 32–109 piRNAs and 400 circRNAs, representing the first characterization of circRNAs in extracellular body fluid 50.

Aside from emerging salivary diagnostics, the use of RNA‐Seq methodology in cancer diagnostics in other body fluids and tissues is very common 45, 51, 52. For example, currently there are known splicing signatures of the three most common types of breast tumours [Triple Negative Breast Cancer (TNBC), non‐TNBC and HER2‐positive cancers] identified by means of RNA‐Seq 51. Also, alternative breast cancer 1 (BRCA1) transcripts have been detected in a subset of patients with breast cancer and a family history of breast and/or ovarian cancer 53. Furthermore, diagnosis of acute myeloid leukaemia can be currently made based on the detection of genetic abnormalities such as t(8;21)(q22;q22) translocation or runt‐related transcription factor 1 (RUNX1) fusion RUNX1–RUNX1T1 52.

The recent progress in RNA‐Seq technologies have a potential for exRNAs to serve as non‐invasive diagnostic indicators of the disease in risk assessement, early diagnostics, prognostics and therapeutics for various diseases, including cancers and infectious diseases 45.

### Liquid biopsy

Liquid biopsy is a biofluid test (of matrices such as serum, urine, saliva) that detects circulating tumour cells or circulating cell‐free tumour DNA (ctDNA) shed into the bloodstream by cancer cells undergoing apoptosis or necrosis. Those tests are much more practical compared to genotyping of tumour tissue, which has significant limitations such as tumour heterogeneity, invasiveness, difficulties with sampling and fact that tumour tissue acquired through a biopsy reflects the condition only at the time of the examination 54, 55, 56, 57. In addition, tumour‐associated mutations detectable in various body fluids provide the information about the early detection, assessment of molecular heterogeneity of general disease, its prognosis, recurrence, monitoring of tumour dynamics and the success or failure of systemic therapies 40, 54, 57. Prediction of prognosis in patients with curable cancer disease can already be achieved in several tumours such as breast cancer, melanoma, ovarian or colon cancers 40, 57. Liquid biopsy permits less invasive means of assessing the oncogenic mutation profile of a patient, and can guide the use of targeted molecular therapies resulting in an improvement of clinical outcome in oncological patients 40.

Analytical strategies to detect and quantify ctDNA in bodily fluids include next generation sequencing (NGS), PCR‐based technology, digital PCR, mass spectrometry (MS), denaturing high performance liquid chromatography, peptide nucleic acid (PNA)‐mediated PCR and PNA‐locked nucleic acid PCR clamp, amplification refractory mutation system, beads, emulsion, amplification and magnetics (BEAMing) or pyrophosphorolysis‐activated polymerization 40, 55, 56 (Table 1).

**Table 1:** Major analytical strategies to assess oncogenic mutations from biofluid samples 40

Analytical strategy	Oncogenic mutations	Body fluid	Tumour	Author
Molecular detection platforms for liquid biopsy				
Beads, emulsion, amplification and magnetics (BEAMing)	PIK3CA	Plasma	Colorectal cancer	Tabernero *et al*. 58
	KRAS			
	BRAF			
Polymerase chain reaction (PCR)‐based techniques				
Quantitative reverse transcription PCR (RT‐qPCR)	BRAF, KRAS	Plasma	Colorectal cancer	Spindler *et al*. 59
	KRAS	Plasma	Lung cancer	Freidin *et al*. 60
Droplet digital PCR (ddPCR)	KRAS	Plasma	Colorectal cancer	Taly *et al*. 61
Next generation sequencing	TP53, PIK3CA	Cell‐free plasma	Breast cancer	Nakauchi *et al*. 62
PCR enhancement techniques for liquid biopsy				
Allele specific primer amplification	KRAS, BRAF	Serum or plasma	Colorectal cancer	Thierry *et al*. 63
Enzyme based digestion of sequences	EGFR	Lung pleural fluid	Lung cancer	Asano *et al*. 64
Preferential homoduplex formation assay (PHFA)	1DH1	Serum, cerebrospinal fluid	Glioma	Chen *et al*. 65
	APC	Plasma	Colorectal cancer	Diehl *et al*. 66
Clamped‐based PCR technique	EGFR	Plasma	Non‐small cell lung cancer (NSCLC)	Kim *et al*. 67

### Electromagnetic field‐based methods

According to the literature, the electromagnetic field can be a very useful tool in diagnosis and treatment of cancers 68, 69, 70. Cormio *et al*. performed the study aimed to determine the diagnostic accuracy of non‐invasive electromagnetic detection of bladder cancer by the tissue‐resonance interaction method (TRIM‐prob) with its overall sensitivity, specificity, positive and negative predictive values as well as diagnostic accuracy of 97.9%, 89.9%, 86.8%, 98.6%, and 93.6%, respectively. They concluded that TRIM‐prob bladder scanning could be used to screen asymptomatic patients at high risk of developing a bladder cancer 70. Also, surface plasmon resonance can be implemented for cancer diagnosis and photothermal therapy, in which plasmonic gold nanoparticles contribute to evoke strong electromagnetic fields on the particle surface, thus enabling tumour detection 69. In addition, Zimmerman *et al*. have reported that intrabuccal administration of 27.12 MHz radiofrequency electromagnetic fields, which are amplitude‐modulated at tumour‐specific frequencies, results in detecting as well as blocking the growth of tumour cells in a tissue‐ and tumour‐specific fashion in patients with various forms of cancer 68. Interestingly, there is currently an increased interest in the use of salivary diagnostics in early detection of cancers by means of electromagnetic field, such as lung cancer 71, 72.

Electromagnetic phenomena can be highly useful for diagnostic techniques, but if applied improperly, the electromagnetic field may also cause serious harmful effects such as leukaemia 73, 74 brain tumour 74, 75, breast cancer 74, 76, 77. This depends on the intensity of the applied electric and magnetic fields, the time of exposure as well as the nature and the frequency of changes 78, 79. Because of the large variety of these factors, a wide spectrum of different scientific research studies and their applications are currently undertaken to elucidate the usage of electromagnetic phenomena for cancer diagnostics.

**Figure 1:**
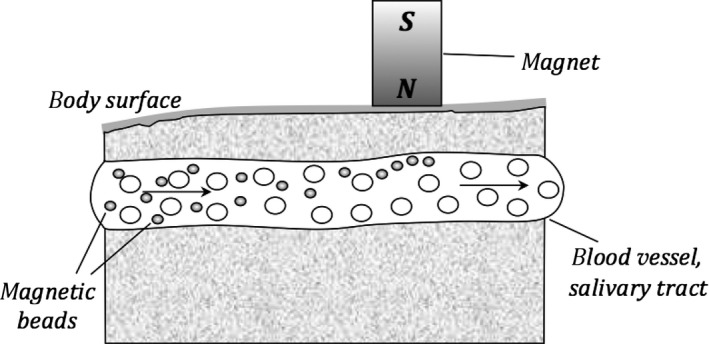
The diagram shows the separation mechanism of selected cells in body fluids by means of ‘magnetic beads’ coated with monoclonal bodies (*in vivo*).

**Figure 2:**
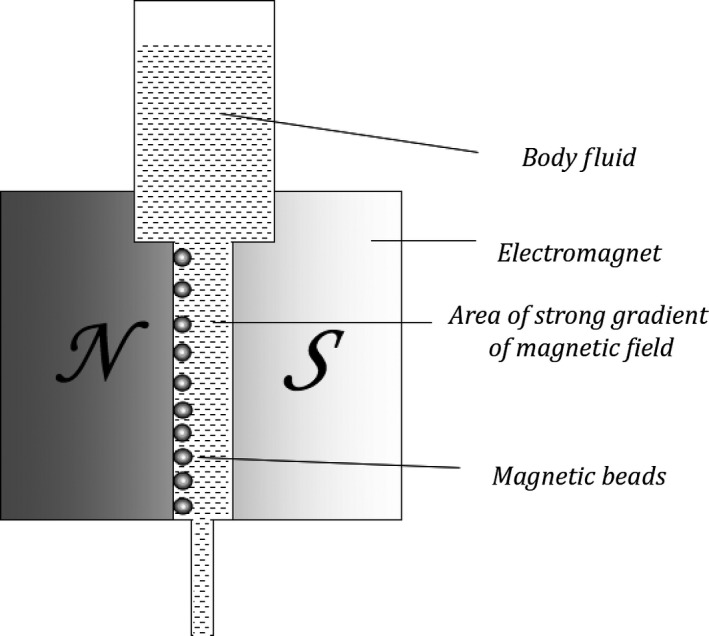
Diagram of magnetic separation of selected components of body fluids using super‐paramagnetic elements (*i.e*. magnetic beads) (*ex vivo*).

One particularly compelling branch where electric and magnetic fields are applied is in the study of exosomal vesicles (EVs), which have a diameter of approximately 30–100 nm. In case of EVs, magnetic beads proved to be very useful in various medical applications including cancer diagnostics 80, 81. Magnetic beads coated with monoclonal antibodies directed against antigens of the specific cells can be easily separated in a magnetic field. They constitute a powerful tool for the isolation of cells, and in particular of exosomes 82. The schemes of such cell separation in body fluids *in vivo* and *ex vivo* are presented in Figures 1 and 2, respectively.

### Electric field induced release and measurement method

The electric field induced release and measurement method (EFIRM) is a newly developed technology at UCLA. EFIRM is an electrochemical‐based technique that measures the oxidation and reduction rates of a chemical reaction to perform quantification of a target biomolecule 28, 38. This is similar to the principles used for traditional glucose metres, which measure the oxidation and reduction rates of glucose oxidase reacting with glucose.

**Figure 3:**
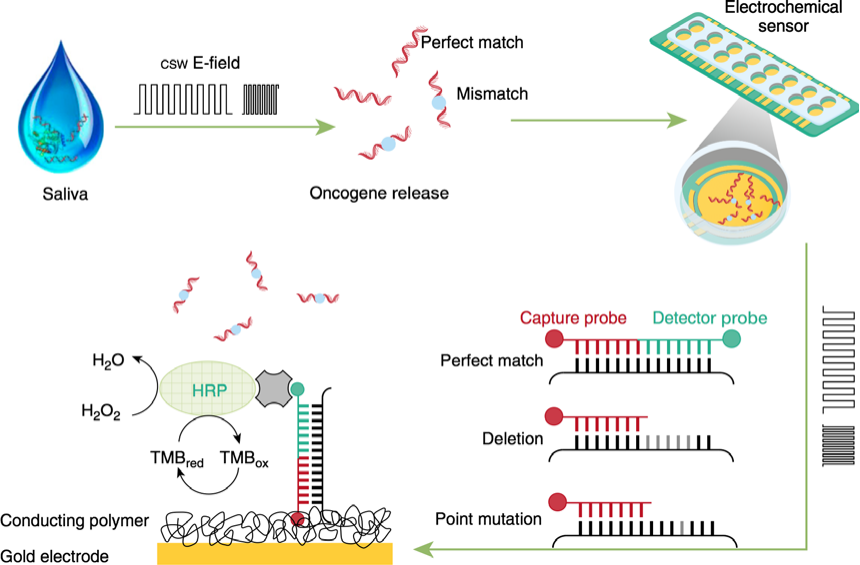
Electric field–induced release and measurement (EFIRM) technology for the detection of epidermal growth factor receptor (EGFR) mutations in bodily fluids of patients with lung cancer (Reproduction from 84).

**Figure 4:**
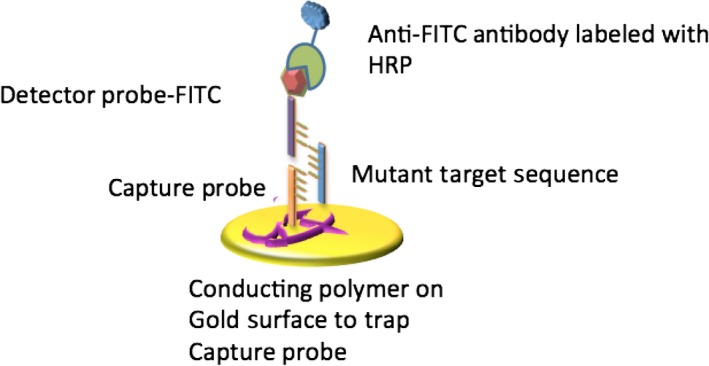
Electric field–induced release and measurement (EFIRM) technology. Following the incubation of the target sequence to the capture probe, a detector probe that is also complementary to the ctDNA is hybridized. The FITC located on the terminal end of the detector probe is then complexed to an anti‐FITC antibody with horseradish peroxidase (HRP).

In case of EFIRM, a capture probe that is complementary to a ctDNA target is designed and then immobilized on the surface of a gold electrode by encapsulating it in a conducting polymer matrix 83. After the immobilization of the capture probe on the surface of the electrode, the clinical specimen (*i.e*. saliva or plasma) is placed on the surface of the electrode and a cyclic square wave (CSW) is applied. This CSW is designed to specifically lyse the exosomal structure that encapsulates the ctDNA sequence and aid in the DNA hybridization process 28 (Fig. 3). Following the incubation of the target sequence to the capture probe, a detector probe that is also complementary to the ctDNA is hybridized. This detector probe has fluorescein isothiocyanate (FITC) located at its terminal end, that is then complexed to an anti‐FITC antibody with horseradish peroxidase (HRP) (Fig. 4).

Finally, the quantification of the amount of the target DNA is performed by adding 3,3′,5,5′‐Tetramethylbenzidine (TMB) and hydrogen peroxide readout substrate mix to the surface of the electrode. Reactions between the HRP enzyme, TMB and hydrogen peroxide will occur, and electronic circuits will be interfaced with the electrode to measure the magnitude of the reaction. If there is a large amount of ctDNA present, then, there will be a high electric current, but if no ctDNA is present, no significant amounts of electrochemical current will be measured.

The EFIRM technique was first deployed in 2009 for the examination of salivary biomarkers for oral cancer detection in a collaborative project between the UCLA School of Dentistry and UCLA School of Engineering 38. More recent work on EFIRM with this platform on detecting ctDNA EGFR mutations in saliva has demonstrated near perfect clinical sensitivity and specificity for detection of lung cancer 38, 56, 84. In a blinded pilot study, 40 patient saliva samples were analysed using EFIRM and compared to tissue‐based oncogenic analysis. Characterizing the performance of EFIRM (area‐under‐the‐curve of 0.94 and 0.96 was achieved for detecting exon‐19 deletion and the L858R mutations, respectively). A comparison of saliva with plasma samples showed R values of 0.98 and 0.99 for the exon‐19 deletion and L858R mutation, respectively 40. Another clinical application of the method that is currently investigated is the detection of oncogenic KRAS gene mutations in patients diagnosed with pancreatic cancer 40.

## Conclusions

A wide variety of emerging saliva‐based technologies have already demonstrated their credibility in the early detection of many cancerous diseases. It is evident that the trend will develop towards constant improvement of POC diagnostic tools, NGS methods, advanced PCR‐ and electromagnetic field‐based technologies as well as liquid biopsy. Further research studies will reveal which method will be the most suitable to be applied in a clinical practice.

## Conflicts of interest

David Wong is co‐founder of RNAmeTRIX Inc., a molecular diagnostic company. He holds equity in RNAmeTRIX, and serves as a company Director and Scientific Advisor. The University of California also holds equity in RNAmeTRIX. Intellectual property that David Wong invented and which was patented by the University of California has been licensed to RNAmeTRIX. Additionally, he is a consultant to PeriRx. None of the other authors have a conflict of interest in relation to the study.
